# Supplementary material for: Effects of Electric-Toothbrush Vibrations on the Expression of Collagen and Non-Collagen Proteins through the Focal Adhesion Kinase Signaling Pathway in Gingival Fibroblasts
Source: Biomolecules. 2022 Jun 1;12(6):771. doi: 10.3390/biom12060771 (PMC9221308; doi:10.3390/biom12060771)
Supplement: Supplementary file 1 [file biomolecules-12-00771-s001.zip › biomolecules-1748393-SI.pdf]

Supplementary Figure S1

<Bioflex plate>

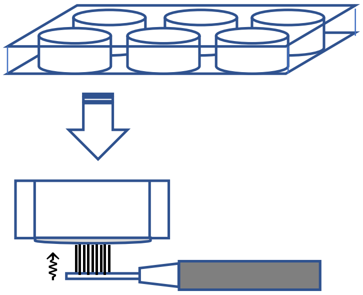

・The plate and toothbrush were each fixed in place, andThe nylon bristles of an electric toothbrush (Gum Care Brush) were placed perpendicularly to the outer surface of the flexible bottom of the Bioflex plate to maintain constant pressure.

・The thickness of the BioFlex® culture plate is 0.5 mm, and the oral epithelium is also approximately 0.5 mm, although there is some variation.

Supplementary Table S1

PCR primers used in the experiments.

Target	Forward primer<br>Reverse primer	Genbank acc no.
Col-1	5'-AGGACAAGAGGCATGTCTGGTT-3'<br>5'-TTGCAGTGGTAGGTGATGTTCTG-3'	NM_000088.4
Col-3	5'-TCCTTGCTGTGGTGGTGTTG-3'<br>5'-GGCAAAACCGCCAGCTT-3'	NM_000090.4
elastin	5'-GGGTTGTGTCACCAGAAGCA-3'<br>5'-CAACCCCGTAAGTAGGAATGC-3'	NM_001081753.3
FN	5'-GATAAATCAACAGTGGGAGC-3'<br>5'-CCCAGATCATGGAGTCTTTA-3'	NM_001365522.2
GAPDH	5'- TGAACGGGAAGCTCACTGG-3'<br>5'-TCCACCACCCTGTTGCTGT -3'	NM_001357943.2

Supplementary Table S2

siRNA target sequences.

siRNA	Target sequence	Target gene
siGAPDH	5'-GUCAACGGAUUUGGUCGUA-3'	h-GAPDH
siPTK2	5'-UAGUACAGCUCUUGCAUAU-3'	h-PTK2
